# Simulated Microgravity Induces Rapid Dielectric Shifts in Erythrocytes of Pancreatic Cancer Patients

**DOI:** 10.1021/acsnanoscienceau.5c00130

**Published:** 2026-01-07

**Authors:** Sai Deepika Reddy Yaram, Alexa Bostic, Ashley Smalley, Soumya K. Srivastava

**Affiliations:** Department of Chemical and Biomedical Engineering, 195919 West Virginia University, Morgantown, West Virginia 26506, United States

**Keywords:** simulated microgravity, pancreatic cancer, PDAC, dielectrophoresis, red blood cells, erythrocytes, space medicine, cancer biomarker, dielectric signatures, adenocarcinoma

## Abstract

Pancreatic ductal adenocarcinoma (PDAC) is among the most lethal cancers, often diagnosed at advanced stages due to the absence of early symptoms and reliable screening tools. Recent studies suggest that systemic changes in cancer, including inflammation, metabolic alterations, and circulating biomarkers, can affect red blood cells (RBCs) or erythrocytes. Although RBCs lack nuclei, those from cancer patients exhibit measurable biophysical and biochemical alterations, offering potential for novel diagnostic approaches. However, it is not known how simulated microgravity might further modulate these cancer-associated RBC dielectric properties, and the effects of microgravity on RBCs from PDAC patients remain largely unexplored. In this study, we investigate the impact of simulated microgravity (SMG) on the dielectric properties of human red blood cells from pancreatic ductal adenocarcinoma (PDAC) patients and healthy donors as controls. Cells were exposed to SMG using a clinostat for 3 and 6 h in a suspending medium with a fixed conductivity of 0.01 S/m. Dielectrophoresis (DEP), a label-free electrokinetic technique, was used to analyze cellular responses to nonuniform electric fields ranging from 0.5 kHz to 45 MHz at a constant peak-to-peak voltage of 10 *V*_pp_. Significant biophysical changes were observed as early as 3 h of SMG exposure. Specifically, a statistically significant increase in specific membrane conductance (*G*_spmem_) was obtained (*p* < 0.0001), and both the force of maximum positive DEP (*F*_pDEP_) and the second crossover frequency (*f*_*x*__o2_) exhibited consistent downshifts, suggesting reduced cytoplasmic conductivity and altered membrane capacitance. Receiver operating characteristic (ROC) analysis indicated that specific membrane capacitance (*C*_spmem_) provides moderate discriminative performance as a candidate SMG-responsive biomarker. These findings demonstrate that RBCs from PDAC patients undergo rapid dielectric changes under simulated microgravity, and underscore the utility of DEP for noninvasive, real-time monitoring of cancer-related biophysical alterations, with applications in both cancer research and space medicine.

## Graphical abstract

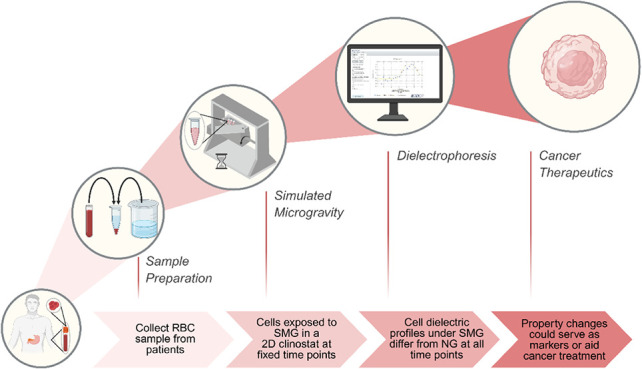

## 1 Introduction

The pancreas is an organ composed of two distinct cell types: exocrine cells, which secrete digestive enzymes into the gastrointestinal tract, and endocrine cells, which release hormones into the bloodstream. Medically, the pancreas is significant due to its role in two major diseases: diabetes mellitus and pancreatic cancer.^1^ Pancreatic cancer, in particular, carries an inferior prognosis, with incidence rates closely paralleling mortality rates.^2^ According to the World Cancer Research Fund (WCRF), pancreatic cancer was the 12^th^ most common cancer globally in 2022, with 510,992 new cases. It ranks as the 11^th^ most common cancer in both men and women. Countries such as Japan, France, and Hungary report the highest age-standardized rates (ASRs). In the United States, there were 60,127 new cases in 2022, with an ASR of 8.6 per 100,000 people. Pancreatic cancer is highly aggressive, with a five-year survival rate of just 13%. Due to the absence of early symptoms, the disease is often diagnosed at advanced stages, significantly contributing to its high mortality rate.

Gravitational force plays a critical role in shaping the physiology and functions of all living organisms on Earth. Since the first human orbital flight in 1961,^3^ the significance of gravity has become increasingly evident. As space exploration extends beyond low Earth orbit, understanding how altered gravity affects biological systems is more important than ever. Exposure to microgravity (10^–6^ g) and varying gravitational levels, such as those on the Moon (0.16 g) and Mars (0.37 g), will become increasingly common. True zero gravity can only be achieved in space, with most research conducted aboard the International Space Station (ISS) under near weightless conditions.^4^ However, conducting research in space presents numerous challenges, including the need for specialized equipment to endure extreme conditions, high costs, difficulties in isolating microgravity effects, and long preparation, operation, data collection, and validation timelines.^5^ To address these constraints, ground-based platforms such as drop towers, sounding rockets, and parabolic flights have been developed to simulate microgravity, but still require funding and infrastructure. Alternatively, laboratory-scale devices such as clinostats, rotating wall vessels (RWV), random positioning machines (RPM), and diamagnetic levitation systems offer cost-effective and adaptable solutions. These technologies can also be integrated with lab-on-a-chip (LOC) systems to enhance experimental efficiency.^6^ It has long been recognized that the absence of gravity exerts harmful physiological effects on the human body. Astronauts returning from extended missions experience muscle atrophy, bone density loss, impaired immune function, and decreased neurological performance.^7–11^ Recently, the impact of microgravity on cancer biology has emerged as a critical area of investigation. Studies suggest that microgravity can impair immune cell function and disturb homeostasis, potentially increasing cancer risk.^12–15^ While both normal and malignant cells exhibit altered behavior in microgravity, the underlying mechanisms remain unclear. Further research is needed to determine whether microgravity could offer insights into new cancer therapies, although its clinical application remains a long-term prospect.

Adding to this growing body of evidence, Pereira-Veiga et al. observed that red blood cells (RBCs) from breast cancer patients exhibited a distinct protein profile compared to cancer-free controls. Additionally, variations in the RBC proteome were correlated across different tumor stages, suggesting that these changes could potentially be used as noninvasive biomarkers for the diagnosis and progression of breast cancer.^16^ Cui et al. investigated the predictive values of RBC-related indicators in patients with resectable gastric cancer (GC). Their study revealed that lower pretreatment mean corpuscular hemoglobin concentration (MCHC) levels were associated with poorer overall survival (OS), while higher MCHC levels predicted better OS. Treatment, including surgery and chemotherapy, resulted in significant changes in mean corpuscular hemoglobin concentration (MCHC), mean corpuscular volume (MCV), and red blood cell distribution width (RDW). Other RBC-related indicators did not show a significant correlation with outcomes.^17^

The study of RBCs in cancer patients is increasingly recognized as essential, as alterations in the RBC proteome and associated hematologic indicators can offer valuable insights into cancer prognosis and progression. Continually circulating throughout the body, RBCs are exposed to specific antigens, tissue degradation products, and other disease-related factors influencing their biophysical and biochemical characteristics. Consequently, their properties reflect the physiological state of the host and the presence of a disease.^18^ These alterations hold promise as noninvasive biomarkers for early cancer diagnosis, disease monitoring, and predicting treatment effectiveness. Their accessibility and dynamic responsiveness offer a simpler, less invasive alternative to traditional diagnostic approaches, positioning RBCs as a valuable tool in precision oncology.

Red blood cells or erythrocytes play a vital role in gas exchange.^19^ They are rich in hemoglobin (HGB), a protein composed of four globin subunits (two α and two β chains) each surrounding an iron-containing heme group, which gives RBCs their red color and enables O_2_ and CO_2_ transport.^20^ RBCs comprise ∼84% of the total blood cell count in a typical adult.^21^ Common RBC-related indicators include RBC count, hemoglobin (HGB), hematocrit (HCT), mean corpuscular volume (MCV), mean corpuscular hemoglobin (MCH), mean corpuscular hemoglobin concentration (MCHC), and RDW.^17^ Anemia, characterized by a deficiency of RBCs, is a frequent complication in cancer patients.^22^ HGB concentration is a well-established prognostic indicator in squamous cell carcinoma of the esophagus,^23^ head and neck.^24^ HCT, which measures the percentage of blood volume occupied by RBCs, can also indicate anemia.^25,26^ MCV reflects the size of RBCs and may signal folate deficiency^27^ while MCH denotes the average hemoglobin content per cell, correlating with iron metabolism.^28^ MCHC, calculated by dividing HGB by HCT, provides insights into the hemoglobin concentration within RBCs.^29^ RDW quantifies the size variability among RBCs, aiding in the diagnosis of different types of anemia.^30^ RBC membranes are in constant interaction with body tissues and are affected by metabolic and microcirculatory factors.^18^ Alterations in the membrane phospholipids and rheological properties during cancer progression have been proposed as diagnostic biomarkers.^31,32^ Several studies have reported an increased cholesterol content, leading to adaptive changes that reduce membrane fluidity.^33–35^ Alterations in cholesterol and total phospholipid levels can disrupt membrane permeability, ion transport, and enzyme activity, ultimately affecting cellular homeostasis.^36–38^

In this study, RBCs from pancreatic cancer patients were analyzed using a powerful electrokinetic technique, Dielectrophoresis (DEP). DEP, first described by Pohl,^39^ is a phenomenon within “AC-electrokinetics” (AC = Alternating Current, electro = electric, kinetics = force), where neutral matter moves due to polarization in a nonuniform electric field.^40–44^ It allows noninvasive measurement of cell electrophysiological parameters, such as conductivity (charge-carrying ability) and permittivity (charge-storage ability) of the cytoplasm and membrane, by observing frequency-dependent DEP collection rates.^45–52^ Dielectrophoresis has been previously employed as a powerful technique for investigating red blood cells (RBCs)^44,53–57^ and has found significant applications in cancer research.^58–64^ The application of dielectrophoresis in these contexts has facilitated advancements in biophysical studies and therapeutic strategies.

This study is novel in integrating DEP, simulated microgravity (SMG), and RBCs from pancreatic cancer patients, a combination not previously explored, and includes comparisons with RBCs from healthy individuals to demonstrate that the observed effects are specific to pancreatic cancer. Significant alterations in the dielectric properties of red blood cells were observed after exposure to SMG. In particular, specific membrane conductance emerged as a promising marker reflecting the sensitivity of RBC membranes to gravitational changes. These results demonstrate the potential of DEP as a powerful, label-free technique for detecting subtle biophysical alterations in cells. This approach holds promise not only for advancing cancer diagnostics but also for understanding cellular responses in space medicine.

## 2 Theory

Dielectrophoresis (DEP) is an electrokinetic phenomenon in which polarizable particles, such as cells including erythrocytes, experience a net force when exposed to a nonuniform electric field while suspended in a medium to which an electric current has been applied through electrodes.^56^ This movement is induced independently of the net charge of the particle and is dependent on the interaction between the particle’s induced dipole and the spatial gradient of the electric field. Dielectrophoresis is most commonly observed in experimental setups involving nonuniform microchannels, which are specifically engineered to generate a nonuniform electric field to enhance bioparticle manipulation capabilities. Dielectrophoresis has broad applications across scientific and academic fields, enabling the label-free separation, concentration, and characterization of living cells and molecules. This technique provides a powerful platform for diagnostics and cell sorting without the need for labeled markers or tags.^65^

For the dielectrophoretic manipulation of bioparticles to occur, a force must be generated to facilitate their movement. This phenomenon arises from the interaction between the nonuniform electric field and the polarizable particles, forming the basis for diverse applications in biophysics and biomedical engineering. The force is quantified by the equation:

1 $$F_{DEP}=4\pi r^{3}\varepsilon_{0}\varepsilon_{m}Re\left[k\left(w\right)\right]\left|E^{2}\right|\approx Re\left[k\left(w\right)\right]V_{rms}^{2}$$

where *F*_DEP_ is the dielectrophoretic force, *r* is the particle radius, *ε*_0_ is the permittivity of vacuum, *ε_m_* is the medium’s permittivity, *E* is the electric field, *Re*[*k*(*w*)] is the real part of the Clausius–Mossotti factor, and *V* is the rms voltage.^66^ By utilizing these parameters, the magnitude of the force exerted by dielectrophoresis on various polarized cellular structures can be quantitatively assessed. This analytical approach allows for a deeper understanding of the interactions between electric fields and biological cells, contributing to advancements in fields such as biophysics and biomedical engineering. The Clausius-Mossotti factor is expressed as a function of permittivities of the cell and the medium they are suspended in as in eq 2.

2 $$k\left(w\right)=\frac{\varepsilon_{\mathrm{cell}}^{*}-\varepsilon_{\mathrm{medium}}^{*}}{\varepsilon_{\mathrm{cell}}^{*}+2\varepsilon_{\mathrm{medium}}^{*}}$$

$$where\;\varepsilon_{cell}^{*}=\varepsilon_{cell}-\frac{j\sigma_{cell}}{\omega },\varepsilon_{medium}^{*}=\varepsilon_{\mathrm{medium}}-\frac{i\sigma_{\mathrm{medium}}}{\omega }$$

where, *k*(*w*) is the polarizability factor, which utilizes the cell permittivity, $\varepsilon_{cell}^{*}$ ; and medium permittivity, $\varepsilon_{medium}^{*}$ , which are involved in the force that the electric field is capable of imparting to the particle. The polarizability factor also utilizes angular frequency, ω, and electrical conductivity of the cell, *σ*_cell_.^67^ Permittivity refers to the ability of a particle or medium to become polarized in response to an electric field. Furthermore, a substance with greater permittivity has a greater ability to be polarized. These molecules become polarized as their dipole moments realign to oppose the applied electric field, generating a net force that induces their movement.^68^

The Clausius-Mossotti factor relies on the membrane capacitance and the conductivity of the cytoplasm, properties found in all cell types and described by the single-shell model, as shown in Figure 1. This model provides an accurate framework to interpret the dielectrophoretic behavior of cells. It represents a living cell as a simplified spherical structure with a dielectric core (cytoplasm) surrounded by a dielectric shell (plasma membrane). RBCs exemplify this single-shell model.^69^ RBC membranes consist of a lipid bilayer, which forms the outer shell; they do not contain any interior organelles, maximizing hemoglobin content and enabling their passage through narrow capillaries. The relationship between the cytoplasmic core and membrane shell in this single shell model is shown in eq 3 where $\varepsilon_{cyto}^{*}$ is the cytoplasm permittivity and $\varepsilon_{mem}^{*}$ is the membrane permittivity.^70^

3 $$\varepsilon_{cell}^{*}=\varepsilon_{cyto}^{*}\left(\frac{1+2\beta }{1-\beta }\right)\;where\;\beta =\frac{\varepsilon_{mem}^{*}-\varepsilon_{cyto}^{*}}{\varepsilon_{mem}^{*}+2\varepsilon_{cyto}^{*}}$$

**Figure 1:**
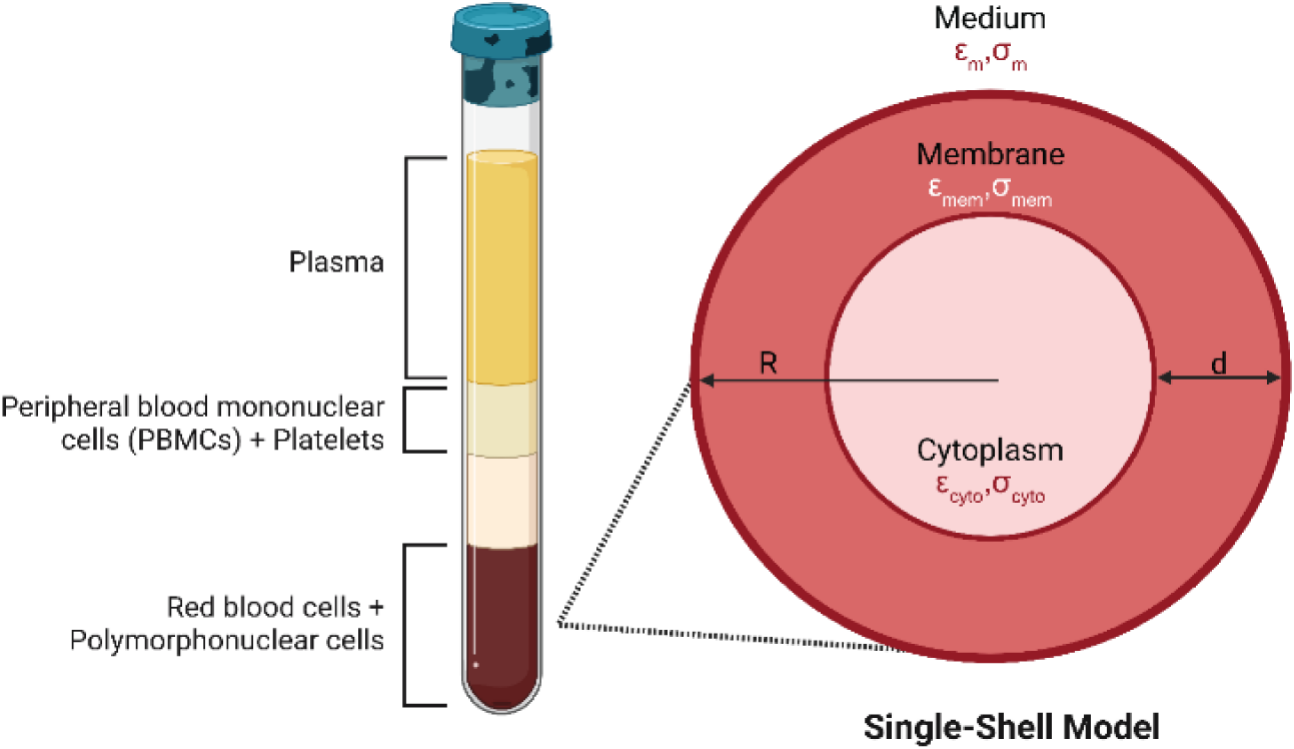
Composition of whole blood and dielectric single-shell model of RBCs representing permittivity (*ε*) and conductivity (*σ*) of cell membrane (mem), cytoplasm (cyto), and suspending medium (m).

Dielectrophoresis occurs as two phenomena: positive dielectrophoresis (pDEP) and negative dielectrophoresis (nDEP), both dependent on the cell and surrounding medium conductivities. The distinction between pDEP and nDEP is indicated by the sign of the real part of the Clausius–Mossotti Factor in the DEP force (eq 1). When this value is positive, the particles are drawn toward the electrode, showing pDEP; and when negative, the particles are repelled from the electrode, exhibiting nDEP. A positive real part occurs when the cell conductivity is greater than the medium conductivity, while a negative value occurs when the cell conductivity is less than the medium conductivity.^44^

The crossover frequency is the specific frequency at which the real part of the Clausius–Mossotti factor equals zero, marking the transition point between pDEP and nDEP or *vice versa*, as shown in eq 4

4 $$f_{ox}=\frac{\sigma_{medium}}{\sqrt{2}R\phi \pi C_{membrane}},\;\mathrm{where}\;\phi =\frac{A}{{\left(3V\sqrt{4\pi }\right)}^{2/3}}$$

where, *f_ox_* is the crossover frequency, σ_medium_ is the conductivity of the medium, *R* is the cell radius, ϕ is the folding factor (with *A* as membrane area and *V* as the cell volume), and *C*_membrane_ is the effective membrane capacitance. The effective membrane capacitance is the interaction of the cytoplasm and the surrounding medium across the membrane as modeled by the single-shell model.^71^ The folding factor combines the idea of the smooth shell that is used within the single shell model and considers the realistic state of a rigid cell membrane lipid bilayer.^64^ While in this state, a particle will experience no movement within an electric field gradient as the net dielectrophoretic force is zero.

Many studies utilizing dielectrophoresis employ 3DEP platforms that incorporate a three-dimensional (3D) electrode configuration within the microdevice to generate the nonuniform electric fields required for cell manipulation and characterization. To achieve the multidimensional electric field that reaches vertically as well as horizontally, these platforms contain connecting electrodes on the glass base of the device, paired with a conductive top electrode. With this configuration, the DEP forces acting on the cells are enhanced, which then allows for more precise cell characterization using their dielectric properties, including membrane capacitance and cytoplasmic conductivity.^71^ In a previous study by K. Hoettges et al., such a 3DEP system was used to characterize RBCs. They obtained a whole-cell capacitance of 1.32 ± 0.02 pF from DEP data, which was comparable to the literature presented, which showed a capacitance between 1.3 and 1.6.^72,73^ Their findings validate the platform’s ability to accurately identify dielectric properties.

## 3 Methods

### 3.1 Red Blood Cell Extraction and Suspension

Blood samples were obtained in collaboration with the Biospecimen and Translational Research Analysis Core (BioTRAC), following written informed consent and compliance with Institutional Review Board (IRB# 2406990595) guidelines at the School of Medicine, West Virginia University, from patients diagnosed with Pancreatic Ductal Adenocarcinoma (PDAC) and healthy people. Nine patients aged over 70 (four females and five males) were enrolled in the PDAC group of this study, and 9 biological samples were collected from healthy individuals. Patient identifiers and relevant clinical details were recorded. Approximately 5 mL of whole blood was collected from each participant via venipuncture. Whole blood (1 mL) was collected from the top of the blood collection tube and transferred to a 1.5 mL Eppendorf tube. Samples were centrifuged at 2500 rpm for 15 minutes at room temperature (RT) using a tabletop centrifuge. The plasma and white blood cell layers were carefully removed and discarded, leaving only the packed red blood cell (RBC) pellet. The pellet was washed by resuspending in 1 mL of 0.9% NaCl, followed by centrifugation at 2500 rpm for 10 minutes. The supernatant was discarded, and the RBC pellet was retained.

Prior to the dielectric experiments, the RBCs were diluted in a suspending medium prepared using 100 mL of deionized water, 8.6 g of sucrose, and 0.306 g of dextrose anhydride. The conductivity of the solution was adjusted to 0.01 S/m using 1× Phosphate Buffered Saline (PBS). The cell suspension was adjusted to the proper concentration by adding the concentrated RBCs to the suspending medium. Once suspended, the cell concentration was adjusted based on images produced during trial runs from the 3DEP software (DepTech, Uckfield, U.K.).

### 3.2 Microgravity Experimental Setup

Microgravity conditions were simulated using a 2D clinostat, in which samples are rotated perpendicular to the axis of gravity to prevent particle settling and create a “simulated microgravity environment” (SMG).^74,75^ For this experiment, the clinostat rotates on a horizontal axis at a controlled speed of 50–60 rpm, driven by a stepper motor controlled by a Digital Stepper Driver (DM542T) and regulated via an Arduino. This setup yields an adequate gravitational acceleration of approximately 10^–3^ – 10^–2^ g. Human RBC samples were suspended in 1.5 mL centrifuge tubes containing a fixed-conductivity medium (0.01 S/m) and placed in the clinostat for 0, 3, or 6 h. Normal gravity (NG – 1g) control samples were handled identically but kept stationary under the same environmental conditions. Temperature (T) was monitored throughout the experiments for both the SMG and NG groups, and no significant variation was observed due to rotation (T = RT), indicating that the observed cellular responses were attributable to altered gravity rather than thermal effects.

### 3.3 Dielectric Properties Characterization

Changes in cellular dielectric properties induced by microgravity are assessed using dielectrophoresis, a technique that enables real-time monitoring of cellular state without the use of biochemical labels.^47,50,76,77^ Prior to microgravity exposure, the dielectric profile of the RBCs (controls) is obtained using the 3DEP software. The cell suspension is then subjected to microgravity conditions, and dielectric profiles are collected at each fixed time point. To preserve the microgravity-induced state, post exposure measurements are taken immediately after clinorotation ends, minimizing any re-exposure to normal gravity.

The 3DEP system utilizes a chip containing 20 microwells, each embedded with electrodes that generate a specific electric field to expose the cells to dielectrophoretic conditions. During the experiment, red blood cells (RBCs) were exposed to 20 varying frequency points ranging from 1 kHz to 45 MHz across the microwells. Cellular responses were analyzed by fitting their behavior to the Clausius–Mossotti polarizability factor using image capture and analysis software. For data analysis, frequency bands 5–9 were selected, and the peak-to-peak voltage (*V*_pp_) was set to 10. The cell suspension was loaded into the microwells, covered with a glass slide, and inserted into the 3DEP analyzer. Only runs with DEP curve fit R^2^ values above 90% were included, yielding a total of 9 data points (*n* = 9), based on nine biological replicates. A schematic overview of the experimental setup is shown in Figure 2.

**Figure 2:**
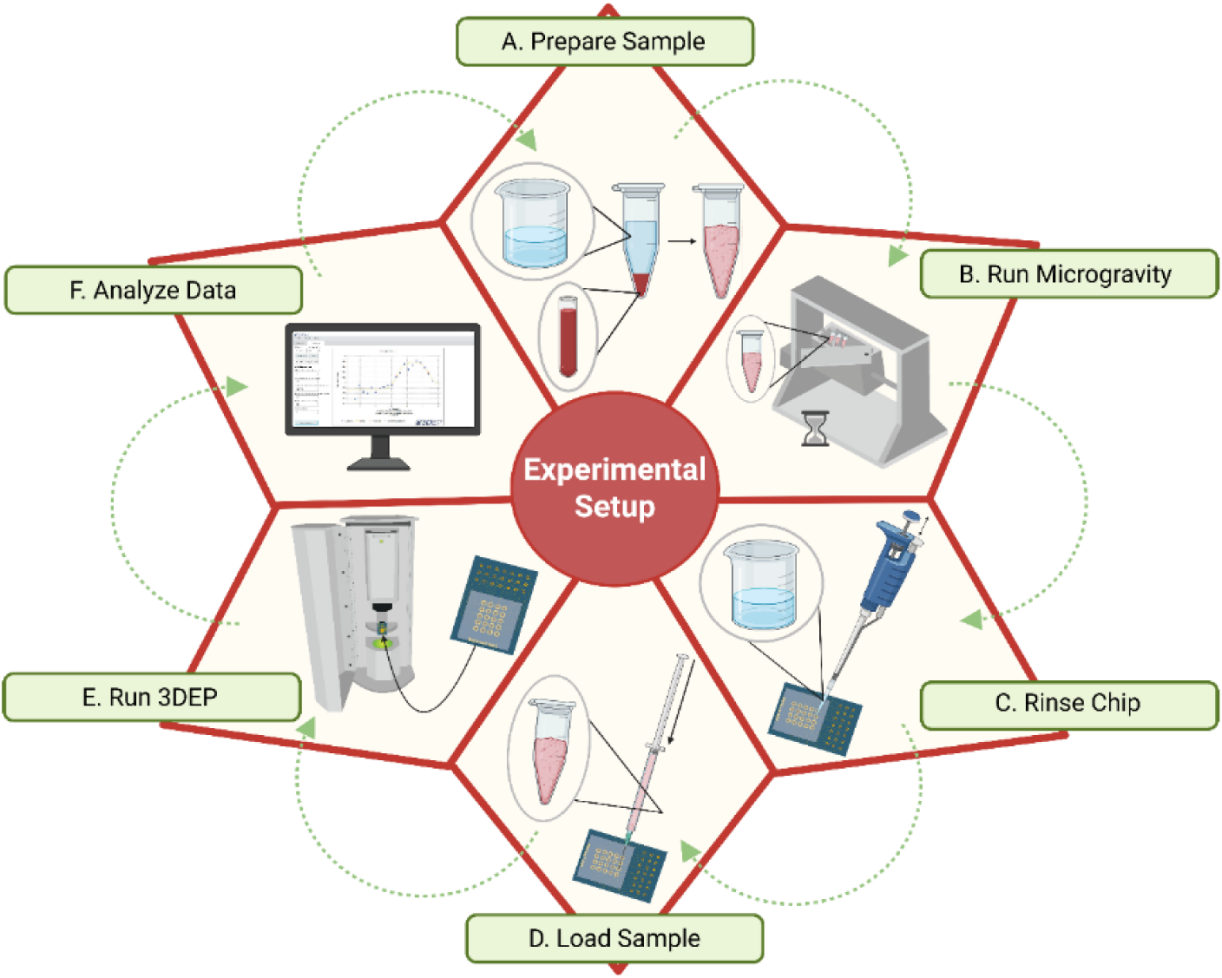
Schematic overview of the experimental workflow. The experimental setup consisted of six main steps: (A) Fresh whole blood samples were processed to isolate red blood cells (RBCs) and then resuspended in low-conductivity media, 0.01 S/m. (B) The prepared samples were exposed to simulated microgravity (SMG) using a 2D clinostat, and a few were maintained under normal gravity (NG) conditions for fixed time points (3 and 6 h) for RBCs from both healthy and PDAC samples. (C) The 3DEP chip was rinsed and prepared for measurements. (D) Samples were loaded into the chip for dielectrophoretic analysis. (E) Dielectric properties of the cells were measured using the 3DEP analyzer. (F) The collected data were analyzed to assess changes in cell dielectric properties.

### 3.4 Statistical Analysis

The statistical analysis was conducted using GraphPad Prism software. The dielectric property data, which included cytoplasmic conductivity, specific membrane capacitance, and specific membrane conductance, were tested for normality using the Shapiro–Wilk test since the sample size was less than 50. If either data set deviated from normality, a Mann–Whitney U test was used, whereas if the data were normally distributed, an unpaired *t* test with Welch’s correction was performed to compare, with statistical significance determined by p-value calculations. Bar plots were created to display the mean ± standard deviation (SD). To evaluate the discriminative ability of each dielectric property, Receiver Operating Characteristic (ROC) curves were generated by comparing the measurements taken at the 3-h and 6-h SMG time points to the 0-h control. The area under the ROC curve (AUC) was calculated for each property. Radar plots were constructed in MATLAB to visualize multivariate comparisons across all conditions: 0 h, 3 h NG (normal gravity), 6 h NG, 3 h SMG, and 6 h SMG. The properties included in the radar plots were cytoplasm conductivity, specific membrane capacitance, specific membrane conductance, first crossover frequency, second crossover frequency, and folding factor.

## 4 Results and Discussions

Data normality was assessed using Q–Q plots and the Shapiro–Wilk test (Figure 3A–C), supporting comparative statistical analysis across experimental groups. This study was designed to determine whether dielectric responses to simulated microgravity (SMG) are specific to red blood cells (RBCs) from pancreatic ductal adenocarcinoma (PDAC) patients and to evaluate whether SMG induces time-dependent alterations within PDAC RBCs. The results demonstrate that both conditions are satisfied, as seen in Figure 3D–F and Table 1.

**Table 1:** Statistical Significance of Pairwise Comparisons between Healthy and PDAC RBCs and between Normal Gravity (NG) and Simulated Microgravity (SMG) at the Indicated Time Points (n = 9 for each group)^a^

Comparison	Time (h)	σ_cyto_ (S/m)	*G*_spmem_ (S/m^2^)	*C*_spmem_ (F/m^2^)
Healthy *vs*. PDAC (NG)	0	ns	***	ns
Healthy *vs*. PDAC (NG)	3	*	ns	***
Healthy *vs*. PDAC (NG)	6	ns	ns	*
Healthy *vs*. PDAC (SMG)	3	**	ns	ns
Healthy *vs*. PDAC (SMG)	6	***	*	ns
NG *vs*. SMG (PDAC)	3	ns	ns	*
NG *vs*. SMG (PDAC)	6	*	ns	ns
NG (0 h) *vs*. SMG (3 h), PDAC	3	**	****	***
NG (0 h) *vs*.SMG (6 h), PDAC	6	****	****	**

a Significance is reported for cytoplasmic conductivity (σ_cyto_), specific membrane conductance (*G*_spmem_), and specific membrane capacitance (*C*_spmem_). Significant *p*-values are denoted as * for *p* < 0.05, **for *p* < 0.01, *** for *p* < 0.001, **** for *p* < 0.0001, and *ns* for non significant.

**Figure 3:**
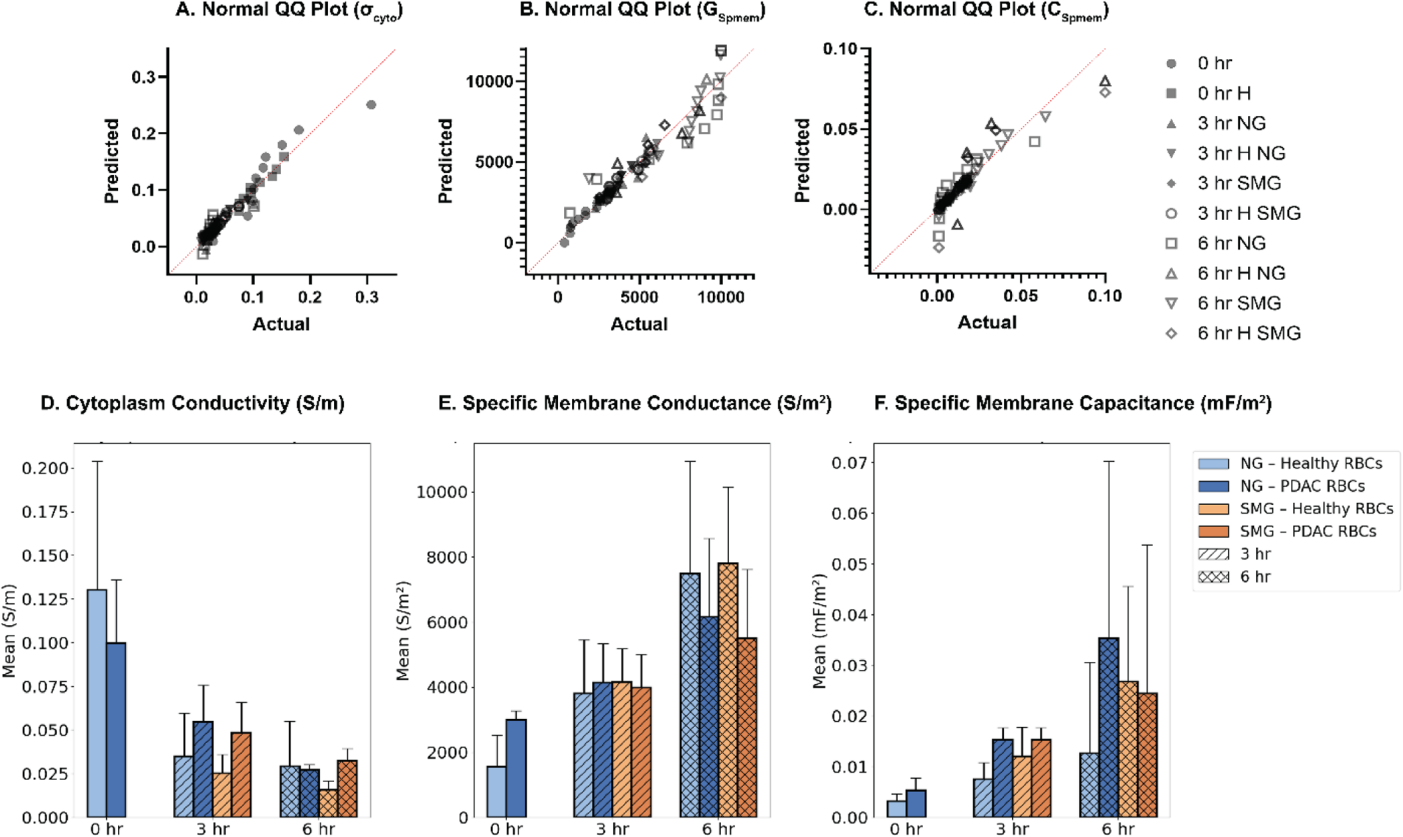
(A–C) Normal Q–Q plots illustrating the distribution of experimental data for cytoplasmic conductivity (σ_cyto_), specific membrane conductance (*G*_spmem_), and specific membrane capacitance (*C*_spmem_) across 0, 3, and 6 h NG and SMG conditions, confirming suitability for subsequent statistical analysis. (D–F) Bar plots showing mean ± standard deviation (SD) values (*n* = 9 for each group) of σ_cyto_ (D), *G*_spmem_ (E), and *C*_spmem_ (F) for RBCs from healthy donors and PDAC patients under 0 h control, 3 h NG, 6 h NG, 3 h SMG, and 6 h SMG conditions. Notably, significant differences between healthy and PDAC RBCs were observed across multiple dielectric parameters and time points, whereas healthy RBCs did not exhibit comparable shifts, indicating that the observed alterations in dielectric properties are primarily associated with PDAC status rather than gravity exposure alone. Pairwise statistical comparisons and corresponding p-values supporting these observations are summarized in Table 1.

First, the dielectric response to SMG was found to be disease-specific. Across all measured parameters, cytoplasmic conductivity (σ_cyto_), specific membrane conductance (*G*_spmem_), and specific membrane capacitance (*C*_spmem_), RBCs from healthy donors exhibited minimal or no significant changes under SMG exposure. In contrast, PDAC RBCs exhibited pronounced, statistically significant alterations, indicating that the observed SMG effects are not intrinsic to RBCs but are amplified by PDAC-associated cellular and membrane remodeling.

Second, SMG induced distinct time-dependent changes in PDAC RBCs. Comparisons across SMG exposure durations revealed progressive, parameter-specific alterations, with stronger effects at 6 h than at 3 h. Cytoplasmic conductivity and membrane conductance exhibited the most robust temporal sensitivity, while membrane capacitance showed earlier but less sustained changes. These findings indicate that PDAC RBCs respond dynamically to prolonged SMG exposure rather than exhibiting a transient or nonspecific stress response.

Collectively, these results establish that SMG-induced dielectric alterations are both PDAC-specific and time-dependent, supporting the conclusion that the pathological state, rather than gravity alteration alone, governs the electrical response of RBCs under simulated microgravity.

Previous studies have reported increased dielectric permittivity (ε′) in the red blood cells of cancer patients, independent of cancer type, potentially due to a thinning of the hydrated shells surrounding the RBCs or structural changes in these shells, which consist of water molecules bound to the cell membranes.^78^ In this study, comparisons between SMG and the 0 h control revealed changes in cytoplasmic conductivity and specific membrane conductance at both the 3 and 6 h time points, with stronger effects at 6 h. These dielectric shifts influence the Clausius–Mossotti plot, as the first crossover frequency reflects the cell size, shape, membrane permeability, and morphology; the positive dielectrophoresis (pDEP) bandwidth is governed by cytoplasmic conductivity and nuclear permittivity; and the second crossover frequency corresponds to the nucleus-to-cytoplasm volume ratio.

The Clausius–Mossotti (CM) plot shown in Figure 4A was generated using MATLAB to illustrate the experimental results. This plot was constructed based on the mean dielectric property values derived from the experiments and interpreted through the single-shell model and equations detailed in the theory section. Interestingly, positive dielectrophoresis (pDEP) was only observed in the 0 h normal gravity (NG) control group. In all other conditions, cells failed to polarize more strongly than the surrounding medium, suggesting possible biological alterations such as membrane disruption or changes in cytoplasmic composition. In PDAC RBCs, the first crossover frequency (*f*_xo1_) is not observed because cancer-associated membrane alterations and elevated cytoplasmic conductivity shift it below the experimental frequency range, suppressing low-frequency positive DEP responses.

**Figure 4:**
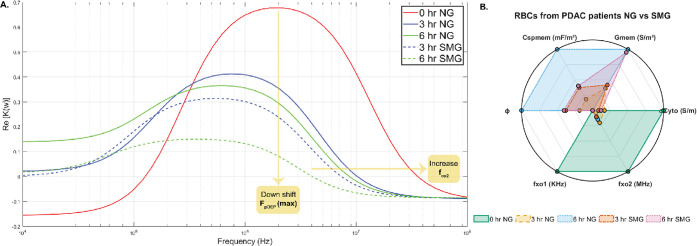
Dielectric profiling of red blood cells (RBCs) under simulated microgravity (SMG) and normal gravity (NG) conditions at 0.01 S/m. (A) Clausius–Mossotti (CM) plots generated using mean dielectric values show a downshift in the frequency of maximum positive DEP (*F*_pDEP_) and the second crossover frequency (*f*_xo2_) under SMG, indicating reduced cytoplasmic conductivity and altered membrane properties. (B) Radar plots compare six key dielectric properties: cytoplasmic conductivity (σ_cyto_), specific membrane capacitance (*C*_spmem_), specific membrane conductance (*G*_spmem_), first crossover frequency (*f*_xo1_), second crossover frequency (*f*_xo2_), and folding factor (ϕ), highlighting distinct dielectric signatures in SMG-exposed cells relative to controls.

Cell viability was confirmed at every time point using the Trypan Blue exclusion assay, indicating that despite these dielectric shifts, the cells remained alive throughout the 6 h duration. The radar plot in Figure 4B, also created in MATLAB using data in Table 2, highlights trends across conditions: 0 h (control), 3 h NG, 6 h NG, 3 h SMG, and 6 h SMG.

**Table 2:** Summary of the Mean Dielectric Properties for Red Blood Cells (RBCs) from PDAC Patients Exposed to Simulated Microgravity (SMG) at 0.01 S/M Medium Conductivity and Fixed Time Points (n = 9)

Properties/Time Points	0 h (control)	3 h NG	3 h SMG	6 h NG	6 h SMG
Cytoplasm Conductivity, σ_Cyto_ (S/m)	0.13	0.03	0.02	0.01	0.03
Specific Membrane Conductance, *G*_spmem_ (S/m^2^)	1564.63	3823.11	4168.72	7802.55	7494.86
Specific Membrane Capacitance, *C*_spmem_ (F/m^2^)	0.0032	0.0075	0.0121	0.0268	0.0127
First Crossover Frequency, *f*_xo1_ (kHz)	126.13	0	0	0	0
Second Crossover Frequency, *f*_xo2_ (MHz)	35.78	10.39	7.38	8.72	4.06
Folding Factor, *ϕ*	0.35	0.84	1.34	2.99	1.41

A consistent downshift was observed in both the frequency of maximum positive dielectrophoresis (*F*_pDEP_) and the second crossover frequency (*f*_xo2_). This shift was primarily attributed to a reduction in cytoplasmic conductivity, likely reflecting decreased mobility of intracellular ions, possibly due to cytoplasmic damage or dehydration. Concurrently, increases in membrane capacitance and conductance suggest changes in membrane structure, such as disrupted lipid organization, altered cholesterol content, or compromised membrane integrity. These changes may also have increased membrane permeability, leading to leakage of intracellular components and further lowering cytoplasmic conductivity.

The downward trend in *f*_xo2_ points to the cell’s diminishing ability to maintain polarization at higher frequencies, causing it to behave more like an insulator. Notably, even small changes in membrane properties can significantly affect this high-frequency response. The lower *f*_xo2_ values observed under SMG indicate altered intracellular conductivity and potential membrane damage, suggesting that microgravity may compromise the cell’s functional capacity to polarize under electrical stress.

Receiver operating characteristic (ROC) curves were generated to evaluate the ability of individual dielectric properties to discriminate SMG-exposed red blood cells (RBCs from PDAC patients) from the 0 h normal gravity control group (Figure 5). Cytoplasmic conductivity (σ_cyto_), specific membrane conductance (*G*_spmem_), and specific membrane capacitance (*C*_spmem_) were assessed at both 3 and 6 h time points. Discriminatory performance was quantified using the area under the ROC curve (AUC).

**Figure 5:**
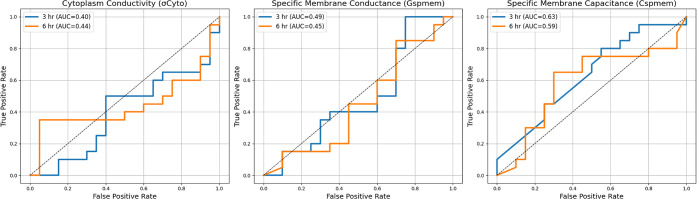
Receiver operating characteristic (ROC) curves were generated to evaluate the ability of cytoplasmic conductivity (σ_cyto_), specific membrane conductance (*G*_spmem_), and specific membrane capacitance (*C*_spmem_) to discriminate between SMG-exposed red blood cells (RBCs) and corresponding 0 h control samples at 3 and 6 h time points. σ_cyto_ exhibited poor discriminatory performance at both 3 h (AUC = 0.40) and 6 h (AUC = 0.44), indicating limited sensitivity to SMG exposure. Similarly, *G*_spmem_ showed near-random classification ability at 3 h (AUC = 0.49) and 6 h (AUC = 0.45). In contrast, *C*_spmem_ demonstrated moderate discriminatory power, with AUC values of 0.63 at 3 h and 0.59 at 6 h, suggesting a comparatively greater sensitivity of membrane capacitance to SMG-induced alterations. The dashed diagonal line represents random classification (AUC = 0.5).

For cytoplasmic conductivity, ROC analysis revealed limited discriminative capability at both time points, with AUC values of 0.40 at 3 h and 0.44 at 6 h, indicating performance below random classification. Similarly, specific membrane conductance exhibited poor discrimination, yielding AUC values of 0.49 at 3 h and 0.45 at 6 h, consistent with no meaningful separation between SMG-exposed samples and controls. In contrast, specific membrane capacitance demonstrated modest but improved discriminatory performance relative to the other properties. AUC values of 0.63 at 3 h and 0.59 at 6 h were obtained, suggesting weak-to-moderate classification capability, though still below the threshold typically associated with robust biomarkers. Notably, the higher AUC observed at 3 h indicates that early SMG-induced membrane remodeling may be more distinguishable on a per-sample basis than later responses.

While bar plot analysis revealed statistically significant group-level differences across multiple dielectric parameters (Figure 3D–F), ROC analysis provided a more stringent, sample-level evaluation of classification performance. The discrepancy between these analyses highlights that statistically significant mean differences do not necessarily translate into effective discrimination at the individual-cell level.

Overall, these findings indicate that none of the evaluated dielectric properties alone provides strong discriminatory power for identifying SMG exposure relative to baseline controls. Among the parameters tested, specific membrane capacitance showed the greatest sensitivity, although limited, whereas cytoplasmic conductivity and specific membrane conductance were ineffective as standalone classifiers. These results suggest that SMG-induced dielectric alterations in PDAC RBCs are subtle and heterogeneous, and that multivariate or combined-feature approaches may be required to achieve reliable discrimination. However, ROC discrimination was limited by sample-level variability and overlapping feature distributions, suggesting that multivariate or integrated dielectric metrics may be required for improved classification performance.

The observed increase in specific membrane conductance (*G*_spmem_) under simulated microgravity (SMG) suggests early alterations in the integrity and permeability of the red blood cell (RBC) membrane, highlighting its potential as a sensitive biophysical biomarker. In RBCs, the plasma membrane plays a crucial role in maintaining ionic homeostasis, mechanical stability, and deformability, primarily through its lipid bilayer and supporting spectrin–actin cytoskeleton.^79^ An increase in membrane conductance reflects a rise in passive ion permeability, which may result from membrane lipid reorganization, mechanical stress-induced alterations, or oxidative damage, all of which have been reported in both cancer and microgravity environments.^80,81^ Unlike specific membrane capacitance, which is largely influenced by membrane surface area, thickness, and dielectric composition, conductance is more directly linked to the function of ion channels, membrane integrity, and the presence of membrane defects.^82^

The absence of significant changes in cytoplasmic conductivity (σ_cyto_) during short SMG exposures is consistent with the lack of intracellular organelles in RBCs and the relatively stable ionic composition of their cytoplasm, which typically changes only in the event of hemolysis or severe osmotic imbalance.^79^ Previous studies have shown the alterations in dielectric properties of RBCs in various disease contexts, such as malaria,^83,84^ babesiosis-infected RBCs,^70^ and separation of human breast cancer cells from blood based on dielectric differences.^85^ It was reported by Gascoyne et al. that stable electrorotation spectra were obtained for normal RBCs, and when analyzed using a single-shell oblate spheroid dielectric model, a plasma membrane-specific capacitance of 12 ± 1.2 mF/m^2^, a cytoplasmic permittivity of 57 ± 5.4, and a cytoplasmic conductivity of 0.52 ± 0.05 S/m were obtained.^83^ Similarly, a specific membrane capacitance of 9 ± 0.8 mF/m^2^ was reported by Becker et al. for healthy RBCs.^85^ In the present study, RBCs isolated from PDAC patients exhibited a significantly lower specific membrane capacitance of 3.2 mF/m^2^, indicating a substantial deviation from the values reported for healthy RBCs. This reduced membrane capacitance in PDAC RBCs could be attributed to alterations in the lipid composition, membrane protein content, or cholesterol levels, which are known to affect membrane dielectric properties. Cancer-associated oxidative stress, metabolic changes, and inflammation may lead to membrane stiffening, lipid peroxidation, or cytoskeletal remodeling, all of which can reduce the membrane’s ability to store charge, thereby lowering its capacitance. Additionally, changes in the hydration shell or loss of membrane flexibility, common in pathological conditions, could also contribute to the observed reduction. We recognize that this work is based on a modest PDAC cohort (*n* = 9 biological replicates), so a portion of the variability likely reflects natural interdonor differences in addition to PDAC-specific effects. RBC properties are also known to be shaped by factors beyond gravity, including metabolic state, oxidative stress, and systemic disease conditions.^44,70^ In this initial study, we therefore focused on a single cell type, one simulated microgravity condition, and short exposure durations (up to 6 h) to establish feasibility. Within this framework, DEP-based dielectric profiling nonetheless reveals clear, disease-associated signatures and demonstrates promise as a complementary, orthogonal approach for detecting subtle biophysical alterations. Building on these proof-of-concept findings, future studies incorporating additional cell types, longer and more varied microgravity exposures, and integration with multiplex biomarker detection workflows will be important to further validate and broaden the translational potential of this approach.

Overall, these findings suggest that measurable alterations in the biophysical properties of RBCs from PDAC patients are induced by exposure to simulated microgravity, primarily through changes in membrane and cytoplasmic characteristics, despite maintaining cell viability. It is suggested that simulated microgravity has a more pronounced impact on RBCs from cancer patients compared to normal gravity conditions, which may assist in further studies regarding therapeutic benefits.

## 5 Conclusions

In this study, the dielectric properties of red blood cells (RBCs) from pancreatic ductal adenocarcinoma (PDAC) patients and healthy donors were systematically characterized under simulated microgravity (SMG) using dielectrophoresis (DEP). Across experiments, SMG exposure produced measurable, time-dependent alterations in specific membrane capacitance, cytoplasmic conductivity, and specific membrane conductance while maintaining RBC viability. Consistent downshifts in the frequency of maximum positive DEP (*F*_pDEP_) and the second crossover frequency (*f*_xo2_) from Clausius-Mossotti analysis point to SMG-induced changes in membrane composition, permeability, and intracellular conductivity.

Although univariate ROC analysis demonstrated that individual dielectric parameters alone have limited power to classify SMG-exposed vs control RBCs, the behavior of these features together indicates that SMG-induced electrical changes in PDAC RBCs are subtle and heterogeneous, and likely multivariate in nature. These observations motivate the use of combined electrical signatures, rather than single metrics, to more fully capture microgravity-associated effects. We note that RBCs undergo continual turnover, with an average lifespan of ∼120 days and clearance of approximately 5 million RBCs per microliter of blood every second;^86^ along with the modest cohort size in this proof-of-concept study (*n* = 9 for each PDAC and healthy group), these factors may contribute to biological variability and underscore the need for larger follow-up cohorts.

Despite these constraints, our findings demonstrate the utility of DEP-based dielectric profiling, coupled with ROC analysis, as a sensitive framework for probing subtle cellular adaptations to altered gravity. This work supports the potential of DEP profiling as a liquid biopsy–style tool for capturing cancer-associated RBC signatures under stress conditions and highlights its relevance for monitoring astronaut health during space missions. The dielectric shifts observed under clinostat-based SMG provide new insight into the biophysical responses of cancer patient cells and establish a foundation for future investigations in true microgravity environments, such as on the International Space Station. Taken together, these results open promising avenues at the intersection of space medicine, cancer diagnostics, and fundamental studies of cellular responses to microgravity.

## Data Availability

The data sets generated during and/or analyzed during the current study are available from the corresponding author upon reasonable request.
